# Effect of occupation on sleep duration among daytime Japanese workers: A cross-sectional study

**DOI:** 10.1097/MD.0000000000028123

**Published:** 2021-12-10

**Authors:** Takeyasu Kakamu, Tomoo Hidaka, Yusuke Masuishi, Hideaki Kasuga, Shota Endo, Midori Sakurazawa, Yukari Munakata, Kimitaka Tajimi, Tetsuhito Fukushima

**Affiliations:** a Department of Hygiene and Preventive Medicine, Fukushima Medical University, Fukushima, Fukushima, Japan; b Koriyama Health Promotion Foundation, Koriyama, Fukushima, Japan.

**Keywords:** daytime worker, lifestyle, occupation, sleep duration

## Abstract

Occupation is one of the factors contributing to the loss of sleep. Although many studies have investigated sleep loss due to irregular and nighttime shifts, the causes of sleep loss in daytime workers remain unknown. The aims of the present study were to determine whether occupation is a dependent factor for sleep duration and whether working status and lifestyle are related to sleep duration.

We examined the health check results of 17,519 (9028 men and 8491 women) workers who had at least 1 health check between the fiscal years 2013 and 2019. We asked about the workers’ occupation, bedtime, dinner time, overtime work, and commuting time, using a self-administered questionnaire at their health check. The occupations were classified into 4 categories: high white-collar, low white-collar, pink-collar, and blue-collar. We conducted a linear regression model and analysis of covariance to investigate the effect of occupation on sleep duration.

As a result of linear regression analysis, bedtime, overtime work and occupation were significantly associated with decreased sleep duration in males, and bedtime, age, and occupation were significantly associated with decreased sleep duration in females. Analysis of covariance revealed that both male and female blue-collar tended to sleep for significantly shorter durations than those in the other occupations.

The results of the current study indicate that sleep duration is affected by occupation. When determining the cause of loss of sleep, medical personnel should consider their patient's lifestyles and how they have been affected by their occupation.

## 1 Introduction

Sleep loss is associated with poor health outcomes, such as diabetes,^[1]^ cardiovascular disease,^[2,3]^ depression,^[4,5]^ and death.^[2]^ Fatigue due to short or insufficient sleep causes daytime sleepiness. The American Academy of Sleep Medicine stated that rideshare companies and other related industries should take appropriate measures to prevent occupational injury due to sleep-related problems.^[6]^ In Japan, subjective sleep problems are significantly associated to occupational injuries.^[7]^ According to the Organization for Economic Cooperation and Development (OECD) report, the average sleep duration in the Japanese population was 7.4 hour, which was the shortest among the OECD member countries.^[8]^ Short sleep and various sleep-related problems are of great concern, especially in Japan.

Determining insufficient sleep is difficult. Subjective sleep tests are commonly used to estimate fatigue due to sleep loss. Berlin questionnaire or Epworth Sleepiness Scale (ESS) is usually used as a screening test; however, some reports suggest that the reliability of subjective sleep tests is not very high compared with that of objective sleep measures such as the apnea-hypopnea index or respiratory disturbance index.^[9]^ Koutsourelakis reported that many patients with severe obstructive sleep apnea recorded a low ESS score,^[10]^ and Fong reported that approximately one-third of patients with severe obstructive sleep apnea syndrome recorded an ESS score of ≤10.^[11]^ Fatigue due to continuous sleep loss does not often appear as a subjective symptom, and thus preventing sleep loss is important. Since no simple method for measuring sleep deprivation has yet been established, efforts to prevent sleep deprivation are required for health management.

Working is one of the causes of reduced sleep. Ikeda et al^[12]^ reported that the average sleep duration in Japanese daytime workers was 6.5 hour, which was 1 hour shorter than that stated in the OECD report. Shift work is one of the largest contributing factors for sleep loss it causes circadian misalignment,^[13]^ resulting in sleep loss.^[14,15]^ In addition to the effects of work on sleep deprivation, the effects of sleep deprivation on work are also serious. Sleep loss causes daytime sleepiness, fatigue, and performance impairment in the workplace.^[16,17]^ Short sleep duration has been reported to significantly impair working performance.^[18]^ These symptoms may lead to occupational injury; thus, sufficient sleep is also important for maintaining workers’ health. Although many studies have investigated sleep loss due to irregular and nighttime shift work, the causalities between sleep loss and occupation in daytime workers are still unknown.

Some previous studies have investigated the relationship between sleep and occupation^[19,20]^; some types of occupations were associated with short sleep duration but these studies included the effect of shift-work. Many amounts of workers are engaged in daytime work so investigating the factors that interfere with the sleep of daytime workers is required.^[21]^ Lifestyle-related factors, such as dinner time and bedtime, are significantly affected by the working environment, which includes working hours, overtime, and commuting time. In addition, the working environment differed significantly from occupation. The aims of the present study were to determine whether occupation is a dependent factor for sleep duration, and whether working status and lifestyle are related to sleep duration.

## 2 Methods

### 2.1 Study design and subjects

**Figure 1:**
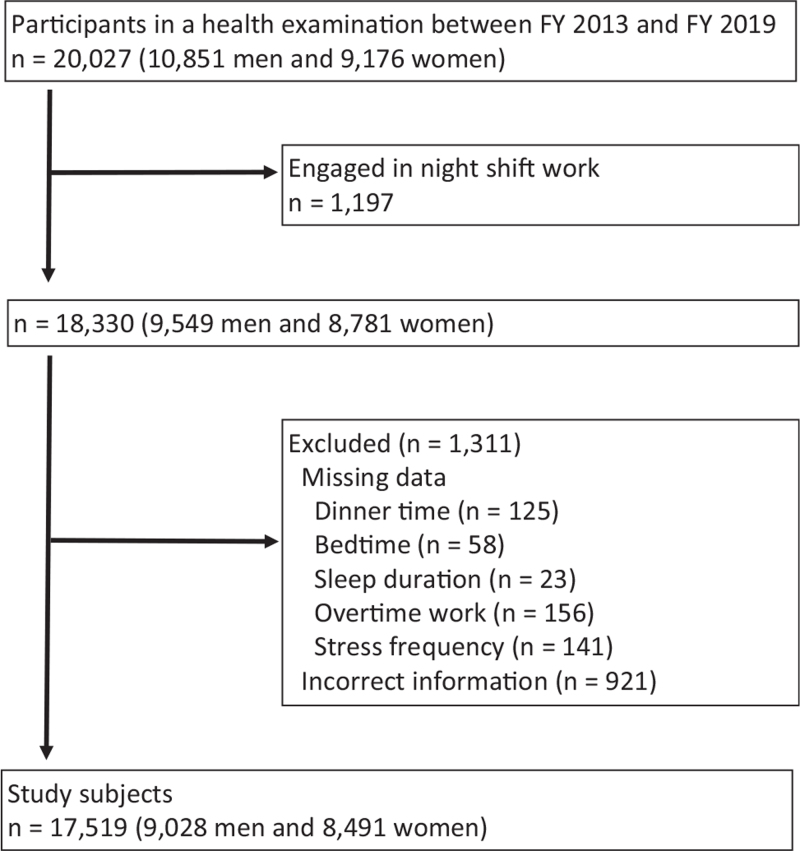
Flowchart of the enrollment of study participants. FY = fiscal year.

This study was designed as a cross-sectional study. Study subjects were participants in a health examination conducted by the Koriyama Health Promotion Foundation based on the Industrial Safety and Health Act between fiscal year 2013 and fiscal year 2019. We included all participants who received at least 1 health check during the study period and agreed to the use of their data. If a participant underwent health checkups twice or more during the study period, we used the data obtained from their first visit. From these participants, we excluded those who engaged in night-shift work and/or had missing data on working status and lifestyle (Fig. 1).

### 2.2 Data collection

In the health checks, we asked questions, using a self-administered questionnaire, on the following topics; dinner time, bedtime, overtime work (h/wk), stress frequency (“how often do you feel stress?” d/wk), commuting time, and occupation (clerks, managers, sales workers, service workers, security workers, teachers, medical workers, agriculture, forestry, and fishery workers, transport workers, manufacturing workers, and construction workers. The occupations were categorized into 4 groups (high white-collar, low white-collar, pink-collar, and blue-collar) according to past studies.^[12,22–24]^ High white-collar workers were engaged in professional work and management work, including manager, teachers, and medical workers^[22,23]^ and low white-collar workers were other white-collar workers including clerks. Pink-collar workers were service sector jobs, including sales, and service workers.^[24]^ Blue-collar included security workers, agriculture, forestry, fishery workers, transport workers, manufacturing workers, and construction workers.^[12,22,23]^ In addition to these questions, we used age and body mass index (BMI) measured at the health check because BMI is known to be associated with sleep.^[25]^

### 2.3 Statistical analysis

We used R 4.0.2 for all statistical analysis. All variables were described as n (%), mean ± standard deviation (SD), and median (25-75 percentile). Student *t* test or chi-square test was used to compare variables by sex. Next, we compared some variables by occupation using analysis of variance and Tukey multiple comparison test. A linear regression analysis was conducted to determine the factors associated with sleep duration. Sleep duration itself was an objective variable, and age, BMI, occupation, overtime work, commuting time, dinner time, and bedtime were explanatory variables. For occupation, low white-collar, was selected as the reference for men, and pink-collar was selected as the reference for women, because the subjects with these occupations had the longest sleep durations in each sex. The coefficient and its 95% confidence interval (95% CI) were calculated and confirmation of multicollinearity of confounding factors were used by variance inflation factor. In this model, a significantly positive coefficient (95% CI > 0) indicates a significant increase in sleep duration, whereas a significantly negative coefficient (95% CI < 0) indicates a significant decrease in sleep duration. After fitting a linear regression model, analysis of covariance (ANCOVA) was conducted to investigate the difference in adjusted sleep duration between occupations. In ANCOVA, sleep duration and occupation were included as a dependent variable and a categorical independent variable, respectively. Age, BMI, overtime work, commuting time, dinner time, and bedtime were included as covariates. Tukey method was used for comparisons between groups.

### 2.4 Ethical approval

All data were anonymized by the Koriyama Health Promotion Foundation. Fukushima Medical University received and analyzed the data for the current study. This study was approved by the Ethics Committee of Fukushima Medical University (Application No. 2020–006).

## 3 Results

During the study period, 20,027 (10,851 men and 9176 women) underwent health checks. We excluded 1197 who engaged in night shift work. Next, we excluded 1311 subjects because of missing data or incorrect information, such as bedtime being earlier than dinner time. Finally, 17,519 study subjects (9028 men and 8491 women) were included in the study.

The mean age ± SD of the study participants was 40.9 ± 12.3 years old. The mean sleep duration ± SD was 6.5 ± 0.9 hour. The characteristics of values according to sex are shown in Table 1. Compared with the females, the males tended to have longer sleep duration (6.6 ± 0.9 hour for males and 6.5 ± 0.9 hour for females, *P* < .001), longer overtime work (6.3 ± 6.7 hour for males and 3.0 ± 4.8 hour for females, *P* < .001), longer commuting time (0.6 ± 0.5 hour for males and 0.5 ± 0.4 hour for females, *P* < .001), later dinner time (19.9 ± 1.2 hour for males and 19.5 ± 1.0 hour for females, *P* < .001), later bedtime (23.5 ± 1.2 hour for males and 23.4 ± 1.0 hour for females, *P* < .001).

**Table 1:** Characteristics.

	Male	Female	*P* value
N	9208 (51.5)	8491 (48.5)	
Age (yrs)	41.4 ± 12.8	40.5 ± 11.8	*<*.001
BMI (kg*/*m^2^)	23.9 ± 3.9	22.1 ± 3.8	*<*.001
Occupation			
High white-collar	3314 (36.7)	4267 (50.3)	*<*.001
Low white-collar	1867 (20.7)	2840 (33.4)	
Pink-collar	2045 (22.8)	1128 (13.3)	
Blue-collar	1802 (19.9)	256 (3.0)	
Overtime work (h/wk)	6.3 ± 6.7	3.0 ± 4.8	*<*.001
Commuting time (h/d)	0.6 ± 0.5	0.5 ± 0.4	*<*.001
Dinner time (pm)	7.9 ± 1.2	7.5 ± 1.0	*<*.001
Bedtime (pm)	11.5 ± 1.2	11.4 ± 1.0	*<*.001
Stress perception (d/wk)	2.4 ± 2.4	2.9 ± 2.4	*<*.001
Sleep duration (h)	6.6 ± 0.9	6.5 ± 0.9	*<*.001

Comparisons of the values by occupation are presented in Table 2. For age, BMI, overtime work, commuting time, dinner time, bedtime, and sleep duration, a significant difference (*P* < .05) was observed by occupation in both males and females. high white-collar (7.5 ± 7.0 h/wk) and low white-collar, (7.1 ± 6.7 h/wk) had significantly longer overtime work in males, and high white-collar, (4.0 ± 5.4 h/wk) had the longest overtime work in females. blue-collar had the shortest overtime work in both males (4.2 ± 6.1 h/wk) and females (1.7 ± 3.3 h/wk). For dinner time, pink-collar (pm 8.5 ± 1.3 for males and pm 7.8 ± 1.3 for females) were the latest, and blue-collar (pm 7.5 ± 1.7 for males and pm 7.3 ± 1.0 for females) were the earliest in both sexes. Sleep duration, in males, was significantly longer in low white-collar, (6.7 ± 0.9 hour) and blue-collar (6.7 ± 1.0 hour) and significantly shorter in pink-collar (6.4 ± 0.9 hour) than the other categories. Stress frequency, in males, was significantly higher in low white-collar, (2.8 ± 2.5 d/wk) and significantly lower in blue-collar (1.9 ± 2.4 hour) than the other categories but no significant difference was observed in females. In the female subjects, low white-collar, (6.6 ± 1.0 hour) had significantly longer sleep duration than high white-collar, (6.5 ± 0.9 hour) and blue-collar (6.4 ± 1.0 hour).

**Table 2:** Characteristics by occupation.

	High white-collar	Low white-collar	Pink-collar	Blue-collar	*P* value
Male					
N	3314	1867	2045	1802	
Age (yrs)	42.5 ± 12.5 a	42.4 ± 12.5 a	38.7 ± 11.6 c	41.1 ± 14.3 b	<.001
BMI (kg/m^2^)	24.1 ± 3.9 a	23.7 ± 3.7 b	23.9 ± 3.9 ab	23.8 ± 3.8 b	.037
Overtime work (h/wk)	7.5 ± 7.0 a	5.2 ± 6.1 b	7.1 ± 6.7 a	4.2 ± 6.1 c	<.001
Commuting time (h/d)	0.6 ± 0.5 a	0.6 ± 0.4 a	0.5 ± 0.5 b	0.6 ± 0.5 a	<.001
Dinner time (pm)	8.0 ± 1.2 b	7.6 ± 1.1 c	8.5 ± 1.3 a	7.5 ± 1.1 c	<.001
Bedtime (pm)	11.5 ± 1.1 b	11.5 ± 1.0 b	11.9 ± 1.2 a	11.1 ± 1.2 c	<.001
Stress perception (d/wk)	2.4 ± 2.3 b	2.4 ± 2.2 b	2.8 ± 2.5 a	1.9 ± 2.4 c	<.001
Sleep duration (h)	6.5 ± 0.9 b	6.7 ± 0.9a	6.4 ± 0.9 c	6.7 ± 1.0 a	<.001
Female					
N	4267	2840	1128	256	
Age (yrs)	41.3 ± 11.8 a	39.5 ± 11.2 b	39.8 ± 12.7 b	42.5 ± 13.9 a	<.001
BMI (kg/m^2^)	22.2 ± 3.8 a	21.8 ± 3.6 b	22.0 ± 3.9 ab	22.6 ± 4.0 a	<.001
Overtime work (h/wk)	4.0 ± 5.4 a	2.2 ± 3.8 b	2.0 ± 4.0 b	0.5 ± 0.5 ab	<.001
Commuting time (h/d)	0.5 ± 0.4 ab	0.5 ± 0.4 a	0.5 ± 0.5 b	1.7 ± 3.3 b	.035
Dinner time (pm)	7.5 ± 0.9 b	7.4 ± 0.9 c	7.8 ± 1.3 a	7.3 ± 1.0 c	<.001
Bedtime (pm)	11.3 ± 1.0 c	11.4 ± 1.0 b	11.7 ± 1.2 a	11.2 ± 1.1 c	<.001
Stress perception (d/wk)	3.0 ± 2.3	2.9 ± 2.3	3.0 ± 2.5	2.7 ± 2.5	.120
Sleep duration (h)	6.5 ± 0.9 b	6.5 ± 0.9 ac	6.6 ± 1.0 a	6.4 ± 1.0 bc	<.001

The results of the multiple linear regression analysis for sleep duration are shown in Table 3. The adjusted *R*^2^ was 0.256 for males and 0.303 for females. All variance inflation factor were under 2. For males, age (coefficient: –0.006, 95% CI: –0.008 to –0.005), BMI (coefficient: –0.012, 95% CI: –0.016 to –0.007), high white-collar (coefficient: –0.110, 95% CI: –0.155 to –0.064), pink-collar (coefficient: –0.087, 95% CI: –0.138 to –0.035), blue-collar (coefficient: –0.237, 95% CI: –0.289 to –0.185), overtime work (coefficient: –0.021, 95% CI: –0.023 to –0.018), commuting time (coefficient: –0.131, 95% CI: –0.164 to –0.098), bedtime (coefficient: –0.365, 95% CI: –0.382 to –0.348) and stress frequency (coefficient: –0.021, 95% CI: –0.028 to –0.013) were significantly associated with increased sleep duration. Bedtime (β = –0.464) and overtime work (β = –0.150) were strongly associated with decreased sleep duration. For females, age (coefficient: –0.016, 95% CI: –0.017 to –0.014), BMI (coefficient: –0.006, 95% CI: –0.011 to –0.002), high white-collar, (coefficient: –0.224, 95% CI: –0.276 to –0.173), low white-collar, (coefficient: –0.130, 95% CI: –0.184 to –0.077), blue-collar (coefficient: –0.365, 95% CI: –0.470 to –0.261), overtime work (coefficient: –0.017, 95% CI: –0.021 to –0.013), commuting time (coefficient: –0.121, 95% CI: –0.160 to –0.081), bedtime (coefficient: –0.489, 95% CI: –0.507 to –0.471) and stress frequency (coefficient: –0.016, 95% CI: –0.023 to –0.009) were significantly associated with decreased sleep duration, and dinner time (β = 0.050) was significantly associated with increased sleep duration. Bedtime (β = –0.544) and age (β = –0.203) were strongly associated with decreased sleep duration.

**Table 3:** Results of multiple linear regression analysis.

	Coefficient (95% CI)	*β*	*P* value
Male (*R*^2^ = 0.256)			
Age	−0.006 (−0.008 to −0.005)	−0.089	<.001
BMI	−0.012 (−0.016 to −0.007)	−0.048	<.001
Occupation (reference Low white-collar)			
High white-collar	−0.110 (−0.155 to −0.064)	−0.057	<.001
Pink-collar	−0.087 (−0.138 to −0.035)	−0.040	<.001
Blue-collar	−0.237 (−0.289 to −0.185)	−0.103	<.001
Overtime work	−0.021 (−0.023 to −0.018)	−0.150	<.001
Commuting time	−0.131 (−0.164 to −0.098)	−0.071	<.001
Dinner time	−0.004 (−0.021 to 0.013)	−0.005	.634
Bedtime	−0.365 (−0.384 to −0.349)	−0.464	<.001
Stress frequency	−0.021 (−0.028 to −0.013)	−0.053	<.001
Female (*R*^2^ = 0.303)			
Age	−0.016 (−0.017 to −0.014)	−0.203	<.001
BMI	−0.006 (−0.011 to −0.002)	−0.025	.006
Occupation (reference Pink-collar)			
High white-collar	−0.224 (−0.276 to −0.173)	−0.122	<.001
Low white-collar	−0.130 (−0.184 to −0.077)	−0.067	<.001
Blue-collar	−0.365 (−0.470 to −0.261)	−0.068	<.010
Overtime work	−0.017 (−0.021 to −0.013)	−0.089	<.001
Commuting time	−0.121 (−0.160 to −0.081)	−0.055	<.001
Dinner time	0.047 (0.028 to 0.066)	0.050	<.001
Bedtime	−0.489 (−0.507 to −0.471)	−0.544	<.001
Stress frequency	−0.016 (−0.023 to −0.009)	−0.040	<.001

The occupational effects on sleep duration as calculated by ANCOVA are shown in Table 4. In males, low white-collar, and pink-collar showed a similar effect on sleep, but the low white-collar, had significantly longer sleep durations than the high white-collar. The blue-collar had a significantly shorter sleep duration than the other occupations. In females, significant differences were observed among all the occupations. pink-collar tended to have the longest sleep duration, and blue-collar tended to have the shortest sleep duration.

**Table 4:** Effect of occupation on sleep duration.

Male	Low white-collar	Pink-collar	High white-collar
Low white-collar			
Pink-collar	0.004		
High white-collar	<0.001	0.889	
Blue-collar	<0.001	<0.001	<0.001

Female	Pink-collar	Low white-collar	High white-collar
Pink-collar			
Low white-collar	<0.001		
High white-collar	<0.001	<0.001	
Blue-collar	<0.001	<0.001	0.022

Source	Sum of squares	df	Mean square	F	*P* value
Male					
Age	59	1	59.1	93.7	<.001
BMI	39	1	38.7	61.4	<.001
Occupation	69	3	23.1	36.7	<.001
Overtime work	388	1	388.0	615.3	<.001
Commuting time	38	1	38.1	60.4	<.001
Dinner time	253	1	252.9	401.1	<.001
Bedtime	1091	1	1090.9	1729.5	<.001
Stress frequency	20	1	19.8	31.4	<.001
Female					
Age	94	1	94.1	161.2	<.001
BMI	21	1	21.0	36.0	<.001
Occupation	10	3	3.3	5.7	<.001
Overtime work	150	1	150.2	257.2	<.001
Commuting time	13	1	12.7	21.7	<.001
Dinner time	160	1	160.1	274.2	<.001
Bedtime	1694	1	1693.5	2900.4	<.001
Stress frequency	11	1	11.1	19.0	<.001

## 4 Discussion

In the present study, we found that there was a difference in sleep duration among various occupations in both males and females. Our statistical model revealed that the occupation was an independent factor affecting sleep duration. Low white-collar had the longest sleep duration, blue-collar had the shortest sleep duration in men and pink-collar had the longest sleep duration, blue-collar had the shortest sleep duration in women. We also assessed the effect of relevant factors on sleep duration and the differences between males and females. Working overtime had a relatively stronger effect on sleep duration in males than in females. Past studies have focused on occupational effects on sleep,^[19,20]^ but they did not consider the effect of shift work, which strongly affects sleep duration and quality.^[13–15]^ In the present study, we focused on workers who did not work night shifts to exclude the effect of shift work. To the best of our knowledge, this study is the first study to focus on daytime workers and occupation is an independent factor affecting sleep duration.

There were gender differences in many items in the present study. Late dinner time was not significantly related to sleep duration in men, but was significantly related to long sleep duration in women. The average sleep duration was 6.6 hour in males and 6.5 hour in females which were similar to those in a past study of Japanese workers.^[12]^ In the present study, about half of the female subjects (49.6%) were categorized as high white-collar. The Koriyama Health Promotion Foundation deals with the health checks of civil servants. Teachers and public health nurses accounted for a large proportion of professionals, and hence the large size of that category. Overtime work was shorter, and dinner time and bedtime were earlier in female subjects. In Japan, many females must cope with a heavy workload at home, including housework and childcare.^[26]^ A 2009 study reported that the time spent on housework by husbands in married couples was only 3 hours per week, which accounted for only 12% of the total housework; the study also reported that the amount was at most 18% when their wives were full-time workers.^[27]^ Because of the heavy workload at home, many working women work jobs with short working hours, or engage in non-regular employment. However, even when husbands spend a short amount of time doing housework, the act of doing housework at all was reported to be satisfactory in many married couples.^[28]^ Many women may feel obligated to do a lot of housework after coming home from their jobs. As a result, this contributes to a shorter overall sleep duration in women than in men.

Overtime work has been reported to be significantly related to decreased sleep duration, especially in males. Overtime work decreases sleep duration, especially on weekdays.^[29]^ The occupation that engages in the most overtime hours is professionals, both in males and females. Professionals include teachers, who have special work systems. Some past study indicated that longer working time and longer commuting time reduce sleep duration.^[30,31]^ Our results support these findings. Reduced sleep duration caused by longer working time and commuting time may lead insomnia and worse health effect^[32]^ so for adequate sleep, it is important to focus on working hours and commuting time.

In males, dinner time in the current study did not have a relationship with longer sleep duration, whereas in females, late dinner time showed a positive relationship with longer sleep duration. Kant et al^[33]^ reported that dinner time is not related to sleep duration. In the present study, sleep duration was related not to dinner starting time but to dinner duration. This reverse correlation may be attributed to some women working long hours eating dinner during working hours. Further studies are needed to investigate the relationship between dinner time and long working hours.

Blue-collar workers had the shortest overtime work, earliest dinner time and earliest bedtime in both men and women, but they also tended to have the shortest sleep duration. Blue-collar workers typically have a large workload and little leisure physical activity^[22]^; the former is reported to decrease sleep quality, whereas the latter increases sleep quality in blue-collar workers.^[34]^ When providing health guidance, medical personnel should ask the workers how they spend their leisure time, which may lead to more informed, effective advice on how to improve their sleeping habits.

Sleep duration was included in the statistical model as a continuous value in this study. Many past studies on sleep durations have defined short sleep duration by setting the cutoff values. Short sleep duration has been commonly defined as <6 hour,^[2,4,35]^ but it has also been reported as <7 hour,^[16]^ <5 hour,^[1]^ and <4 hour^[18]^ in other studies. The present study focused on the differences in sleep duration between occupations. Before the statistical analysis, we performed a normality test and found that sleep duration was normally distributed among the study subjects.

### 4.1 Limitations and strength

The present study has several limitations. First, many of the study participants were civil servants, and teachers were included in the category of professionals. Therefore, a selection bias may have occurred. Second, we did not ask about ways to spend their leisure time, which is as important as their working status to promote good sleep. The strength of this study is that the study subjects were all non-shift workers. By excluding shift workers, we can focus on the labor load itself.

## 5 Conclusions

The results of the present study indicate that sleep duration is affected by occupation. Furthermore, the factors associated with sleep loss differed according to sex. It is important for medical personnel to provide adequate advice and guidance to their patients, taking into consideration the occupation-specific effects of their lifestyle on their sleeping habits.

## Acknowledgments

We would like to thank the Scientific English Editing Section of Fukushima Medical University for English language editing.

## Author contributions

TK and KT conceived the study design. YM and MS managed and anonymized the data. TK and TH conducted the statistical analysis. TK drafted the initial manuscript, and all authors revised the manuscript.

**Conceptualization:** Takeyasu Kakamu, Midori Sakurazawa, Yukari Munakata, Kimitaka Tajimi, Tetsuhito Fukushima.

**Data curation:** Midori Sakurazawa, Yukari Munakata, Kimitaka Tajimi.

**Formal analysis:** Takeyasu Kakamu.

**Investigation:** Takeyasu Kakamu.

**Methodology:** Takeyasu Kakamu.

**Project administration:** Takeyasu Kakamu, Tomoo Hidaka, Yusuke Masuishi, Hideaki Kasuga, Shota Endo.

**Resources:** Takeyasu Kakamu.

**Software:** Takeyasu Kakamu.

**Supervision:** Tomoo Hidaka, Yusuke Masuishi, Hideaki Kasuga, Shota Endo, Kimitaka Tajimi, Tetsuhito Fukushima.

**Validation:** Takeyasu Kakamu.

**Writing – original draft:** Takeyasu Kakamu, Tomoo Hidaka.

**Writing – review & editing:** Tomoo Hidaka, Yusuke Masuishi, Hideaki Kasuga, Shota Endo, Midori Sakurazawa, Yukari Munakata, Kimitaka Tajimi, Tetsuhito Fukushima.
